# EPICANCER—Cancer Patients Presenting to the Emergency Departments in France: A Prospective Nationwide Study

**DOI:** 10.3390/jcm9051505

**Published:** 2020-05-17

**Authors:** Olivier Peyrony, Jean-Paul Fontaine, Sébastien Beaune, Abdo Khoury, Jennifer Truchot, Frédéric Balen, Rishad Vally, Jacques Schmitt, Kasarra Ben Hammouda, Mélanie Roussel, Céline Borzymowski, Cécile Vallot, Veronique Sanh, Elie Azoulay, Sylvie Chevret

**Affiliations:** 1 Department of Emergency Medicine, Saint-Louis University Hospital, Assistance Publique-Hôpitaux de Paris, 1 avenue Claude Vellefaux, 75010 Paris, France; jean-paul.fontaine@aphp.fr; 2 Department of Emergency Medicine, Ambroise Paré University Hospital, Assistance Publique-Hôpitaux de Paris, 92100 Boulogne-Billancourt, France; sebastien.beaune@aphp.fr; 3 INSERM UMRS 1144, Paris-Descartes University, 75006 Paris, France; 4 Initiatives de Recherche aux Urgences (IRU) Research Network, Société Française de Médecine d’Urgence (SFMU), 75010 Paris, France; akhoury@chu-besancon.fr (A.K.); jennifer.truchot@aphp.fr (J.T.); 5 Department of Emergency Medicine & Critical Care, Besançon University Hospital, 25000 Besançon, France; 6 Department of Emergency Medicine, SMUR, Lariboisière University Hospital, Assistance Publique-Hôpitaux de Paris, 75010 Paris, France; 7 Faculty of Medicine, Paris Diderot University, 75010 Paris, France; 8 Department of Emergency Medicine, Toulouse University Hospital, 31059 Toulouse, France; balen.f@chu-toulouse.fr; 9 Faculty of Medicine, Toulouse III—Paul Sabatier University, 31330 Toulouse, France; 10 Department of Emergency Medicine, SAMU 33, Pellegrin University Hospital, 33000 Bordeaux, France; rishad.vally@chu-bordeaux.fr; 11 Department of Emergency Medicine, SAMU 68, Mulhouse Hospital, 68100 Mulhouse, France; jacques.schmitt@ghrmsa.fr; 12 Department of Emergency Medicine, Colmar Hospital, 68000 Colmar, France; kasarra.benhammouda@ch-colmar.fr; 13 Department of Emergency Medicine, Rouen University Hospital, F-76031 Rouen, France; melanie.roussel@chu-rouen.fr; 14 Department of Emergency Médicine, Jean Bernard Hospital, 59322 Valenciennes, France; borzymowski-c@ch-valenciennes.fr; 15 Department of Emergency Medicine, Annecy Genevois Hospital, 74370 Annecy, France; cvallot@ch-annecygenevois.fr; 16 Department of Emergency Medicine, SAMU 95, René Dubos Hospital, 95300 Pontoise, France; vsanh@hotmail.fr; 17 Intensive Care Unit, Saint-Louis University Hospital, Assistance Publique-Hôpitaux de Paris, 75010 Paris, France; elie.azoulay@aphp.fr; 18 Centre of Research in Epidemiology and StatisticS (CRESS), INSERM, UMR 1153, Epidemiology and Clinical Statistics for Tumor, Respiratory, and Resuscitation Assessments (ECSTRRA) Team. University of Paris, 75010 Paris, France; sylvie.chevret@u-paris.fr; 19 Department of Biostatistics and Medical Information, Saint-Louis University Hospital, Assistance Publique-Hôpitaux de Paris, 75004 Paris, France

**Keywords:** cancer, emergency department, epidemiology

## Abstract

Background: We aimed to estimate the prevalence of cancer patients who presented to Emergency Departments (EDs), report their chief complaint and identify the predictors of 30-day all-cause mortality. Patients and methods: we undertook a prospective, cross-sectional study during three consecutive days in 138 EDs and performed a logistic regression to identify the predictors of 30-day mortality in hospitalized patients. Results: A total of 1380 cancer patients were included. The prevalence of cancer patients among ED patients was 2.8%. The most frequent reasons patients sought ED care were fatigue (16.6%), dyspnea (16.3%), gastro-intestinal disorders (15.1%), trauma (13.0%), fever (12.5%) and neurological disorders (12.5%). Patients were admitted to the hospital in 64.9% of cases, of which 13.4% died at day 30. Variables independently associated with a higher mortality at day 30 were male gender (Odds Ratio (OR), 1.63; 95% CI, 1.04–2.56), fatigue (OR, 1.65; 95% CI, 1.01–2.67), poor performance status (OR, 3.00; 95% CI, 1.87–4.80), solid malignancy (OR, 3.05; 95% CI, 1.26–7.40), uncontrolled malignancy (OR, 2.27; 95% CI, 1.36–3.80), ED attendance for a neurological disorder (OR, 2.38; 95% CI, 1.36–4.19), high shock-index (OR, 1.80; 95% CI, 1.03–3.13) and oxygen therapy (OR, 2.68; 95% CI, 1.68–4.29). Conclusion: Cancer patients showed heterogeneity among their reasons for ED attendance and a high need for hospitalization and case fatality. Malignancy and general health status played a major role in the patient outcomes. This study suggests that the emergency care of cancer patients may be complex. Thus, studies to assess the impact of a dedicated oncology curriculum for ED physicians are warranted.

## 1. Introduction

Due to a growing number of new cases and decrease in mortality over recent years, the number of patients with malignancies is expected to increase over the next decade [1]. In France in 2018, there were 382,000 new cases of cancer and 157,400 cancer-related deaths, which represents approximately 29% of all-cause deaths. Currently, 3.7 million individuals (5% of the total French population) are cancer survivors (i.e., individuals who are living with cancer or have a past history of cancer) [2]. These patients are likely to use emergency care resources for medical complications that may reveal malignancy or be due to its treatments or cancer progression [3,4]. Complications may occur even after remission [5]. Descriptive data are therefore needed to both describe how emergency departments (EDs) are attended by cancer patients and report their particular outcomes, such as the need for hospitalization, Intensive Care Unit (ICU) admission and mortality [6]. The Comprehensive Oncologic Emergencies Research Network (CONCERN) [7] identified several research priorities, including the need to collect epidemiologic data. However, only retrospective data based on nationwide survey databases have described the characteristics of the ED visits of patients with cancer [8,9,10]. These studies provided a very comprehensive picture of the reasons why cancer patients visit EDs in the US, but no such results have been reported in Europe. As the number of patients living with cancer is expected to grow in following years, such data might be valuable to better address unmet needs and identify an optimal standard of care for this ED population. Therefore, in a large prospective nationwide study, we aimed to describe the use of French EDs by cancer patients, with a particular emphasis on patients’ characteristics and predictors of 30-day all-cause mortality following hospital admission.

## 2. Patients and Methods

### 2.1. Objectives

The main objectives of this study were to estimate the prevalence of cancer patients that presented to EDs in France, report their chief complaints and describe their characteristics. The secondary objective was to identify the predictors of mortality among the patients admitted to the hospital after ED presentation.

### 2.2. Patients and Study Design

This was a cross-sectional national prevalence study. During three consecutive days, from Tuesday the 6th to Thursday the 8th of February 2018, all EDs in France that consented to participate prospectively included all the consecutive cancer patients they attended.

In France, patients can present to the ED through self-referral or after having called the dispatch center “Services d’Aide Médicale Urgente” (SAMU), where an emergency physician decides the appropriate level of response by sending the patient either an ambulance; the fire department; or a Mobile Intensive Care Unit staffed by an emergency physician, a nurse and a paramedic for pre-hospital medical assistance when a life-threatening condition is suspected. Medical advice can also be provided, or the patient can be referred to a general practitioner or to the ED.

Overall, among all the 622 French EDs, 138 (22.2%) from the Initiative de Recherche aux Urgences (IRU) research network coordinated by the French Society for Emergency Medicine (SFMU) agreed to participate in the study (Table S1). After giving informed consent to participate, all consecutive patients with solid cancer or hematologic malignancy were included, whatever their reason for attending the ED. Exclusion criteria were an age below 18 years and the remission of malignancy for more than five years.

Attending emergency physicians collected standardized data, including demographic data (age, gender); first medical contact prior to ED attendance (none; a non-emergency physician such as a general practitioner, referring oncologist or radiologist; or a SAMU medical dispatch center); reasons why patients were seeking emergency care; underlying malignancy (type and location of the malignancy, time since diagnosis, presence of metastases, disease status); performance status (i.e., a scale developed by the Eastern Cooperative Oncology Group that describes the patient’s ability to care for himself and perform daily activities, ranging from 0 “fully active” to 4 “completely disabled”); presence of life-threatening conditions (shock, acute respiratory or neurological failure according to the attending emergency physician); vital signs at triage, including numerical pain rating scale (NPRS) with a 0 to 10 range, triage level at ED presentation on a 1 to 5 scale (1 being the most severe) [11] and shock-index (heart rate/systolic blood pressure); access to the referring oncologist or the oncologic medical record; in case of a life-threatening condition, the presence of advanced directives (resuscitation or palliative status) in the patient’s medical record; the need to access the referring oncologist’s advice; investigations, interventions and treatments realized in the ED; and hospital admission or discharge after the ED visit. For patients admitted to the hospital from the ED, the assessment included whether they were admitted to ICU, the length of the hospital stay and the 30-day status (discharged, still hospitalized or death). The primary outcome was 30-day all-cause mortality following hospital admission. The study was registered on ClinicalTrials.gov (NCT03393260) and approved by the Institutional Review Board of the French Speaking Society for Respiratory Medicine—Société de Pneumologie de Langue Française (number CEPRO 2017-038).

### 2.3. Statistical Analysis

Patient pathways before ED attendance were described for all patients. Further results only dealt with patients enrolled in the ED. When the same patient was attended by several EDs successively, only the data from the last admission was considered. Descriptive statistics were reported, namely median with interquartile range (IQR) for continuous variables and the number with percentage for binary and categorical variables with a comparison based on the Mann Whitney test or chi square test, respectively. In patients admitted to the hospital, we used logistic regression to identify the predictive factors of all-cause mortality at day 30. Univariable models were first fitted. Then, variables associated with the outcome on the basis of *p*-values less than 0.1 by univariable analyses were selected for a multivariable analysis. When several variables shared a close clinical significance, we only selected variables that appeared more accurate and with the best metrological properties. Clinically relevant variables, such as age or gender, were forced into the model whatever their *p*-value. Then, a backward selection procedure was applied and variables with *p*-values of less than 0.05 were removed. Missing data were imputed, except for the outcome, using multiple imputation by chained equations [12,13]. The multivariable logistic model was applied to the imputed datasets and final estimates were obtained according to Rubin’s rules. The primary analyses of mortality only dealt with patients with available outcomes. Then, sensitivity analyses were conducted; a simple imputation of the outcome (by dead or alive) was performed, as well as simple stochastic imputation based on the observed mortality of 13.4%, assuming outcomes were missing completely at random. All the *p*-values were two-sided, with values of 0.05 or less considered as statistically significant. Data were analyzed with R 3.5.0 software (the R Foundation for Statistical Computing, Vienna, Austria).

## 3. Results

**Figure 1:**
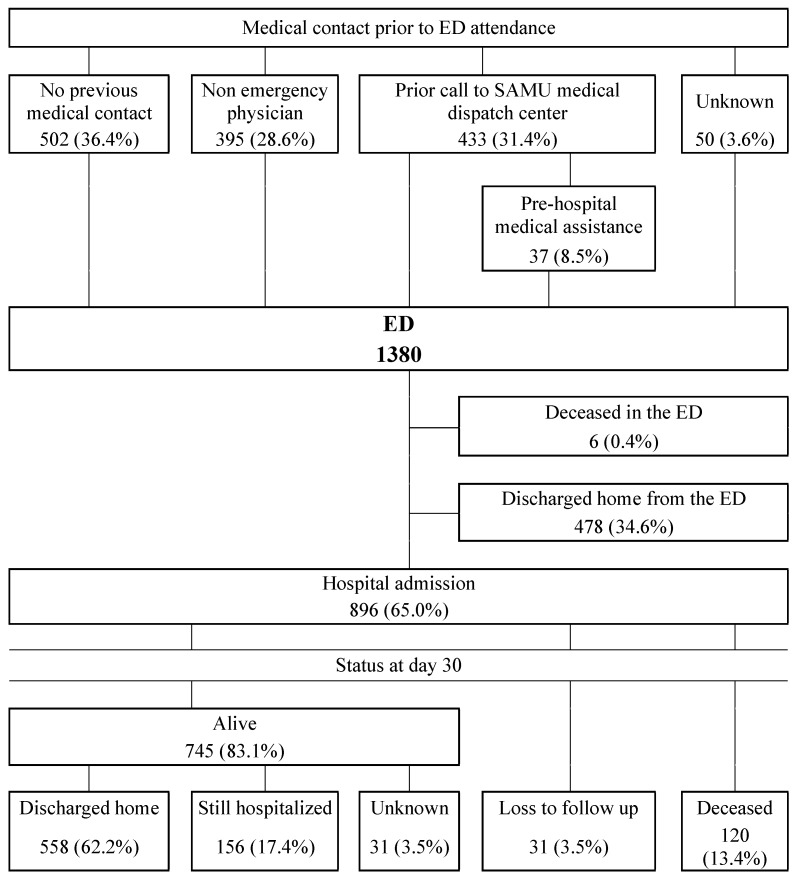
Flowchart of cancer patients attending emergency departments in France throughout the 3-day study period (ED—emergency department; SAMU—Service d’Aide Médicale Urgente).

During the three-day study period, a total of 1380 cancer patients visited the ED in the participating centers. The number of cancer patients included by each center varied greatly from one center to another and ranged from 0 to 40 during these three days, with a median of eight patients per center. The median prevalence was 2.8% (IQR, 1.7%–4%; range, 0–11.1%) (Table 1). The patient trajectories before attending the ED are shown in Figure 1. Among these 1380 patients, 502 (36.4%) presented through self-referral to the ED, 395 (28.6%) were referred by a non-emergency physician to the ED, while 433 (31.4%) first called an emergency physician in a SAMU dispatch center. Of all the patients referred to the ED by a non-emergency physician, 31 (7.8%) were sent by their referring oncologist. Among the 433 patients that first called the SAMU dispatch center, 396 (91.4%) were referred to the ED without pre-hospital medical assistance and 37 (8.6%) with pre-hospital medical assistance.

### 3.1. General Characteristics and Reasons for Seeking Emergency Care

**Figure 2:**
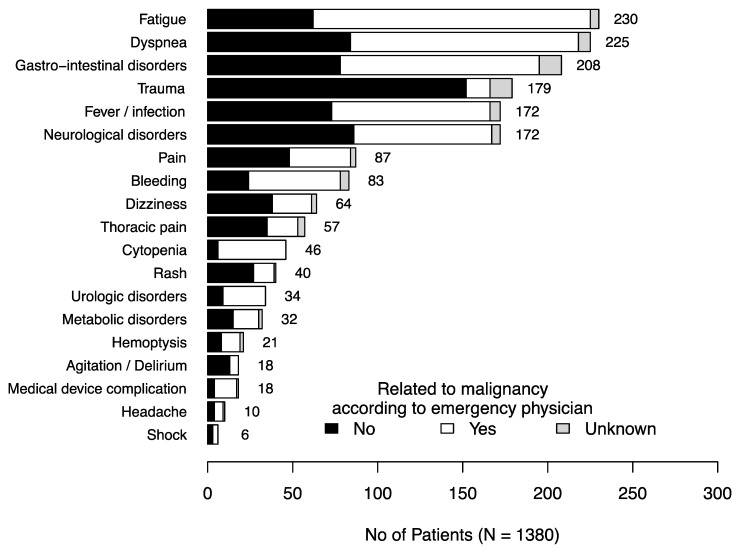
Reasons for cancer patients’ attendance to emergency departments.

The patient characteristics are summarized in Table 2. The patients had mostly solid malignancies (86.1%), were in complete or partial remission (57.0%) and had a good performance status (75.7%). The most frequent reasons why patients sought ED care were fatigue (16.6%); dyspnea (16.3%), gastro-intestinal disorders such as diarrhea, vomiting or abdominal pain (15.1%); trauma (13.0%); fever (12.5%); and neurological disorders (12.5%) (Figure 2). According to the emergency physician in charge of the patient, these reasons were related to the malignancy in 687 cases (52%) but varied depending on the chief complaint (Figure 2).

### 3.2. Presentation at Inclusion

Table 3 shows the cancer patients’ severity, depending on the triage level, the shock-index, the emergency physician’s assessment and the pain assessment at ED arrival.

### 3.3. Access to the Oncologic Medical Record

For 570 (42.7%) of the cases, the patients presented to an ED outside their referring oncology center. The emergency physician had no immediate access to the oncologic medical record of 519 (40.2%) patients. Immediate contact with the referring oncologist was considered unnecessary for 946 (71.7%) patients. Among the 102 patients critically ill at ED admission, no information in the medical record regarding resuscitation or palliative care status was recorded in 65 (69.9%) patients. Among those for whom this information was available, 24 (85.7%) had a palliative care status mentioned in their medical record.

### 3.4. Investigations, Interventions and Treatments

Table 4 reports the investigations, interventions and treatments performed or initiated in the ED. Nearly all the patients (91.8%) had at least one investigation or intervention, such as a blood sample (76.6%) or an X-ray (43.6%). One quarter of the patients underwent a CT-scan. Analgesics (31.2%) and antibiotics (14%) were the most commonly administered treatments. Among the 213 patients with an NPRS ≥ 6, 33 (15.5%) received morphine. The patients received intensive care medications such as fluid challenge, vasoactive agents, non-invasive ventilation, mechanical ventilation or cardiopulmonary resuscitation in 12.6% of the cases. The patients had oxygen therapy in 14.8% of the cases. Among the patients who carried a long-term central venous catheter (CVC) for their chemotherapies, it was used in 13.4% of the cases.

### 3.5. Outcome after ED Visit and at Day 30

**Figure 3:**
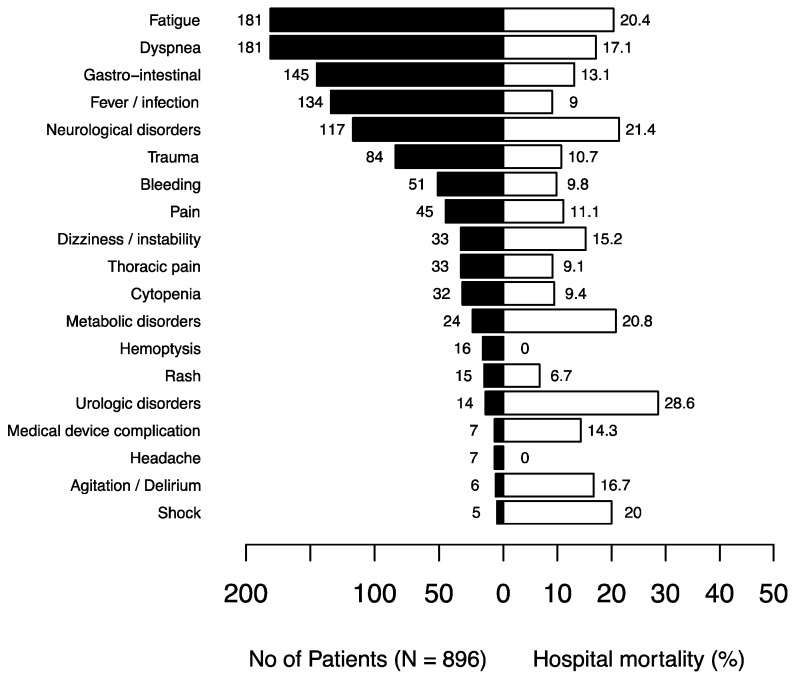
In-hospital mortality rates depending on the reasons for attending emergency departments in hospitalized cancer patients.

As shown in the flowchart (Figure 1), 896 (64.9%) patients were admitted to the hospital and six (0.4%) died in the ED. The median length of stay in the ED was 5 h and 50 min (IQR: 3 h and 45 min to 8 h and 51 min). When admitted to the hospital, the patients were admitted to their referring oncologic department in 176 (19.6%) of the cases and to the ED short stay unit in 229 (25.6%) of the cases. Fifty-nine (6.6%) patients were admitted to the ICU, either (*n* = 38) directly from the ED or during their hospitalization (*n* = 21), one half of whom were admitted during the first three days of their hospital stay. At day 30 after hospital admission, 558 (62.2%) patients had been discharged, 120 (13.4%) had died and 156 (17.4%) were still in the hospital. The median length of hospital stay was 6 (3–12) days. Among the hospitalized patients, Figure 3 shows the rates of in-hospital mortality according to the reasons why they attended EDs. Fatigue; neurological, metabolic and urologic disorders; and shock were the symptoms that carried the highest mortality rate (>20%).

**Figure 4:**
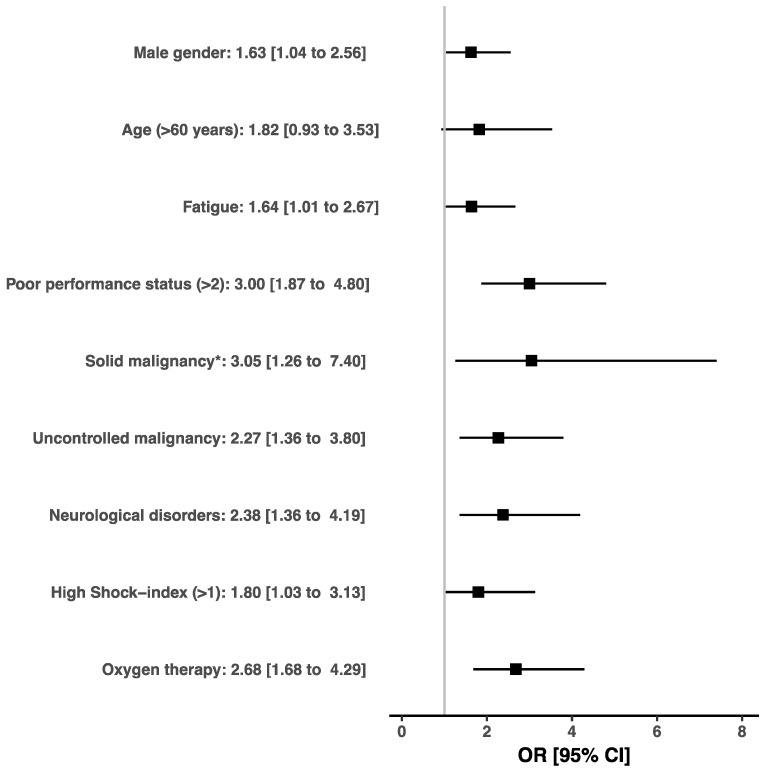
Effects on the 30-day mortality of covariates identified by multivariate logistic regression after imputation of the missing data. * Reference: hematological malignancy.

In hospitalized patients, variables independently associated with a higher mortality at day 30 were male gender (OR, 1.63; 95% CI, 1.04–2.56), fatigue (OR, 1.65; 95% CI, 1.01–2.67), poor performance status (OR, 3.00; 95% CI, 1.87–4.80), solid malignancy (OR, 3.05; 95% CI, 1.26–7.40), uncontrolled malignancy (OR, 2.27; 95% CI, 1.36–3.80), ED attendance for a neurological disorder (OR, 2.38; 95% CI, 1.36–4.19), high shock-index (OR, 1.80; 95% CI, 1.03–3.13) and oxygen therapy (OR, 2.68; 95% CI, 1.68–4.29) (Figure 4). The results of the multivariable logistic model with and without multiple imputation are reported in Table 5.

### 3.6. Sensitivity Analyses

The results of the multivariable logistic models after imputing the 31 missing outcomes were similar, though the influence of the shock-index was no longer significant in the case of imputation by “death”. Fatigue was no longer significant in the case of imputation by “alive” or by stochastic imputation (Table S2).

## 4. Discussion

The first objective of our study was to estimate prospectively the prevalence of cancer patients in French EDs; we found that it was, in median, 2.8%. Even if our study was conducted during a short period and did not concern all French EDs, it should be emphasized that almost one ED in four participated all around the country, providing the first large prospective nationwide prevalence estimate of ED use by cancer patients. This number was close to the 3.4% prevalence reported in a single French ED prospective study in 2007 [14], as well as to those reported in other non-French nationwide retrospective studies [8,9,15]. Based on diagnosis codes, these studies reported a prevalence of ED cancer patients in the US of 4.2% for Rivera et al. [8] and 3.7% for Hsu et al. [9], whereas it was 2.4% for van der Meer et al. in Australia [15]. These nationwide retrospective studies have the benefit of giving a large picture of emergency care use by cancer patients but could underestimate their prevalence. This may be the case when the reason for seeking care is not directly related to the malignancy and cancer is not taken into account when coding the diagnosis. Furthermore, the prevalence may also vary depending on the definition used for inclusion, and terms like “cancer patients”, “active cancer”, “remission” or “cancer-related visit” need to be more accurately defined [6]. We observed that the prevalence varied from one center to another depending on their specificity in onco-hematology, but was somewhat lower than the 5% prevalence of cancer survivorship reported by the French National Institute of Cancer [2]. This could be due to the fact that we chose to include patients with active cancer or who had been in remission for less than five years. Our first result reveals that the number of cancer patients attending EDs every day is substantial and that this requires specific attention from the emergency community with further research on this topic.

The second objective of our study was to describe the reasons why cancer patients sought emergency care. We showed that the most frequent reason was fatigue, followed by potential life-threatening conditions such as dyspnea, neurological disorders and fever. While symptoms like fatigue or gastro-intestinal disorders, which were also frequent, may seem less alarming to emergency physicians, these symptoms could also hamper discharge and participate in the high rate of hospitalization and death among cancer patients after an ED visit [10,16,17]. Indeed, fatigue had a high mortality rate in hospitalized patients and was associated with mortality in the multivariable analysis. In other studies, weakness was also found to be associated with mortality in non-cancer patients in the ED [18,19]. Contrary to other studies in all-comer ED patients [20,21], dyspnea was not associated with mortality in the univariable analysis, whereas this condition was one of the most frequent reasons for cancer patients to attend the ED and had a high mortality rate. More generally, the reasons why patients attended EDs in our study were similar to those found in other studies that focused on ED cancer patients [8,9,10,15,22,23,24], except for trauma, probably because of the inclusion period with snowfalls all across the country. These findings suggest that cancer patients may attend EDs for multiple reasons, either specific to malignancy progression or cancer treatment or due to other non-cancer-related causes. Thus, diagnostic workup can be challenging for a non-experienced emergency physician. Studies focusing on frequent and life-threatening complications in cancer patients, such as febrile neutropenia; dyspnea; or neurological disorders like spinal cord compression, confusion or seizures, are mandatory in the emergency setting [6].

We also sought to describe cancer patients’ pathways before ED arrival, their clinical severity, their management throughout ED attendance and their outcomes. Patients presented mostly through self-referral to the ED or after calling medical dispatch centers. Very few patients were referred by their referring oncologist, leading to the risk that emergency physicians missed out on important information concerning malignancy status and patient prognosis. Moreover, nearly half of the patients attended an ED that was not located in their referring oncologic center, and their oncologic medical record was often not accessible. Surprisingly, emergency physicians declared that contact with the referring oncologist was not necessary in two-thirds of the cases. One patient in ten was described as critically ill and one in four had the highest triage levels at arrival. Very few patients did not have any exam or intervention; most had a blood sample, radiograph, CT-scan or ultrasound and were treated with analgesics, antibiotics or oxygen therapy. A higher use of morphine in patients with high NPRS levels and long-term CVC should be promoted. This high burden of care among these patients illustrates their need for urgent care, consistent with the high rates of hospital admission of 64.9% and in-hospital mortality of 13.4%. This high rate of hospitalization among these patients is similar to those found in other studies that ranged from 29% to 90% [8,9,10,15,23,24]. The mortality rate of 7.9% reported by van der Meer et al. was lower than ours but concerned all ED visits, while ours concerned only hospitalized patients [15]. In this study, patients with cancer-related visits were eight times more likely to die in the hospital than those with visits unrelated to cancer. Vandyk et al. reported in a meta-analysis a median mortality rate of 13%, but this rate varied depending on the timeline, ranging from 1% for mortality in the ED to 56% for mortality at 3 months [24]. Karakoumis et al. prospectively enrolled more than 1200 patients who attended EDs for non-specific complaints during four years and showed that patients with cancer had the highest mortality rate at day 30 (31%), which was six times higher than the overall patient mortality [25].

Finally, our study identified that male gender, fatigue, poor performance status, solid and uncontrolled malignancy, ED attendance for a neurological disorder, high shock-index and oxygen therapy were predictors of 30-day all-cause mortality among hospitalized cancer patients. Mortality was associated with some characteristics inherent to malignancy, such as solid or uncontrolled malignancy, but also with the patient’s general health status such as fatigue as a reason for attending the ED and a dependency level assessed by the performance status. The impact of general health status upon cancer patients’ outcomes may seem obvious and has already been described by other authors [26,27,28]. Nonetheless, it is crucial to take into account these characteristics upon ED management, so as to establish conjointly the goals of care as soon as possible in order to determine the appropriate intensity of care, orientation and monitoring [4]. We also identified clinical prognostic factors for cancer patients, like shock-index or the need for oxygen therapy. If these characteristics are non-specific of patients with malignancies, the use of shock-index at ED triage is very simple and has already proven its utility in recognizing patients at a high risk of complication [29,30,31,32], as is the case with the quick Sequential Organ Failure Assessment score [33]. Hypoxemia is a predicting factor of ICU admission among patients with malignancies, and the level of oxygen delivery has shown to be an early and independent predictor of 28-day mortality in cancer patients admitted to the ICU for acute respiratory failure [34,35]. It should alert emergency clinicians to admit these patients to departments with an adequate level of monitoring.

### Limitations

In order to motivate the highest number of recruiting centers, the dedicated form was short and follow-up was conducted only for admitted patients. Therefore, details about malignancy status, specific treatments, symptoms, ED management and long-term follow-up for the whole cohort were not recorded. Additionally, inclusions took place for three consecutive working weekdays, but a seven-day collection could have given a more thorough picture. Nevertheless, this study gives a general picture of cancer patients visiting EDs in our country. Despite the simple format of the inclusion form, there was missing data, mostly regarding malignancy characteristics or patients’ general health status. Such missing data is consistent with the fact that clinicians lacked specific information concerning cancer status. Likewise, the surprisingly low number of specific treatments in the last three months may be due to missing information and highlights the need for more accessible data in the EDs on patients’ oncologic follow-up. Finally, we cannot exclude non-observed confounding factors.

## 5. Conclusions

This large prospective nationwide study about cancer patients seeking emergency care, who represent 3% of total ED attendance, showed some heterogeneity among reasons for ED attendance and a high need for hospitalization and case fatality. Mortality predictors among hospitalized cancer patients were male gender, fatigue, poor performance status, solid and uncontrolled malignancy, ED attendance for a neurological disorder, high shock-index and oxygen therapy. Malignancy and general health status, information about which is often not available in the emergency setting, both play a major role in patient outcomes, and should be taken into account by emergency physicians to adapt the level of care. This study suggests that the emergency care of cancer patients may be complex. Thus, studies to assess the impact of a dedicated oncology curriculum for ED physicians are warranted.

## Figures and Tables

**Table 1:** Number of inclusions and prevalence of cancer patients in the participating 138 French emergency departments.

Variable		Missing Data
**ED, No**	138	
**Patients Included, No**	1380	
**No of Patients Included by Center**		0
Mean	10	
Median	8	
IQR	4–14	
Max	40	
**Prevalence, %**		17
Mean	3.1	
Median	2.8	
IQR	1.7–4.0	
Max	11.1	

ED—emergency department; IQR—interquartile range.

**Table 2:** Patient characteristics.

Variable		Missing Data (*n*)
**N**	1380	
**Age (Years), Median (IQR)**	71 (61–82)	30
**Female Gender, *n* (%)**	598 (43.6)	8
**Underlying Malignancy, *n* (%)**		4
**Solid Malignancy**	1185 (86.1)	
Digestive and pancreas	223 (18.8)	
Breast	187 (15.8)	
Prostate	175 (14.8)	
Lung	172 (14.5)	
Kidney and bladder	117 (9.9)	
Endometrium and ovary	61 (5.1)	
Head and neck	57 (4.8)	
Skin	55 (4.6)	
Other	138 (11.7)	
**Hematologic Malignancy**	191 (13.9)	
Lymphoma	73 (38.2)	
Chronic leukemia	38 (19.9)	
Myeloma	37 (19.4)	
Acute leukemia	22 (11.5)	
Other	21 (11.0)	
**Time Since Diagnosis, *n* (%)**		122
<6 months	236 (18.8)	
6 months–5 years	753 (59.9)	
>5 years	269 (21.3)	
**Disease Status, *n* (%)**		285
Complete remission	270 (24.7)	
Partial remission	354 (32.3)	
Uncontrolled malignancy	471 (43.0)	
**Metastatic, *n* (%)**	380 (47.1)	573
**Specific Treatments in the Last 3 Months, *n* (%)**		134
Chemotherapy	401 (32.2)	
Hormonotherapy	127 (9.2)	
Radiotherapy	73 (5.9)	
Surgery	65 (5.2)	
Immunotherapy	47 (3.8)	
Other	17 (1.4)	
**Poor Performance Status (>2), *n* (%)**	283 (24.3)	213
**Patient Alone at Home, *n* (%)**	225 (27.7)	569
**Home Nursing Service or Nursing Home Care, *n* (%)**	483 (37.3)	85

IQR—inter-quartile range.

**Table 3:** Clinical severity of cancer patients upon emergency department presentation.

Variables at ED Presentation		Missing Data (*n*)
**N**	1380	
**Critically Ill According to the Emergency Physician, *n* (%)**	102 (8.3)	144
Respiratory failure	44 (3.6)	
Shock	44 (3.6)	
Altered mental status	19 (1.5)	
**High Shock-Index ^a^ (≥1), *n* (%)**	130 (9.9)	71
**Triage Level, *n* (%)**		373
1	50 (5.0)	
2	205 (20.3)	
3	520 (51.6)	
4	200 (19.9)	
5	32 (3.2)	
**Pain Assessment at ED Arrival**	1029 (74.6)	
NPRS ≥ 6	213 (20.7)	

ED emergency department, NPRS numeric pain rating scale, ^a^ Heart rate/systolic arterial blood pressure.

**Table 4:** Investigations, interventions and treatments performed in the EDs for cancer patients.

Variable		Missing Data (*n*)
**N**	1380	
**Exams, *n* (%)**		0
None	113 (8.2)	
Blood sample	1057 (76.6)	
X-ray	602 (43.6)	
CT-scan	358 (25.8)	
ECG	158 (11.4)	
Ultrasound	68 (4.9)	
Other	36 (2.6)	
**Interventions, *n* (%)**		0
Peripheral catheter	1008 (73.0)	
Oxygen therapy	204 (14.8)	
Fluid challenge	164 (11.9)	
Puncture/drainage/catheterization	88 (6.4)	
Management of traumatism	43 (3.1)	
Non-invasive ventilation/mechanical ventilation	11 (0.8)	
Cardiopulmonary resuscitation	1 (0.1)	
**Red Cells or Platelets Transfusion, *n* (%)**	61 (4.4)	
**Medications, *n* (%)**		0
Analgesia	430 (31.2)	
Antibiotics	193 (14.0)	
Morphine	54 (3.9)	
Cardiovascular/coagulation	46 (3.3)	
Nebulization	25 (1.8)	
Sedation/neurological	12 (0.9)	
Vasoactive agents	7 (0.5)	
Other	31 (2.2)	
**Long-Term Central Venous Catheter Carrier, *n* (%)**	243 (23.0)	324
**Use of Long-Term Central Venous Catheter, *n* (%)**	28 (13.4)	34

ED—emergency department.

**Table 5:** Multivariable analysis. Variables independently associated with a 30-day mortality for patients hospitalized after visiting the ED (*N* = 896) before and after the imputation of missing data, except for the outcome that was not imputed (355 patients had at least one missing data, among which 31 concerned the outcome).

	Complete Cases		After Imputation	
	N = 541—Deaths = 81		N = 865—Deaths = 120	
	OR (95% CI)	*p*	OR (95% CI)	*p*
**Male Gender**	1.96 (1.13–3.52)	0.02	1.63 (1.04–2.56)	0.03
**Age (≥60 years)**	1.90 (0.89–4.47)	0.11	1.82 (0.93–3.53)	0.08
**Fatigue**	1.75 (0.97–3.13)	0.06	1.64 (1.01–2.67)	0.049
**Poor Performance Status (>2)**	2.78 (1.63–4.76)	0.0002	3.00 (1.87–4.80)	<0.00001
**Solid Malignancy ^a^**	3.70 (1.41–12.81)	0.02	3.05 (1.26–7.40)	0.01
**Uncontrolled Malignancy**	2.00 (1.14–3.57)	0.02	2.27 (1.36–3.80)	0.002
**Neurological Disorders**	2.69 (1.34–5.28)	0.005	2.38 (1.36–4.19)	0.003
**High Shock-Index ^b^ (≥1)**	2.03 (1.03–3.94)	0.04	1.80 (1.03–3.13)	0.04
**Oxygen Therapy**	2.43 (1.34–4.37)	0.003	2.68 (1.68–4.29)	<0.0001

ED—emergency department; OR—odds ratio; 95% CI—95% confidence interval. ^a^ Reference: hematological malignancy; ^b^ heart rate/systolic arterial blood pressure.

## Supplementary Materials

The following are available online at https://www.mdpi.com/2077-0383/9/5/1505/s1. Table S1: collaborators and members of the Initiatives de Recherche aux Urgences (IRU) research network who participated in the study, Table S2: sensitivity multivariable analyses imputing the missing outcomes either all by death or alive status, or by simple stochastic imputation, assuming missing completely at random outcomes, based on the observed mortality of 13.4%.
