# Supplementary material for: EPICANCER—Cancer Patients Presenting to the Emergency Departments in France: A Prospective Nationwide Study
Source: J Clin Med. 2020 May 17;9(5):1505. doi: 10.3390/jcm9051505 (PMC7291158; doi:10.3390/jcm9051505)
Supplement: Supplementary file 1 [file jcm-09-01505-s001.zip › Table S2.pdf]

Table S2. Sensitivity multivariable analyses imputing the missing outcomes either all by death or alive status, or by simple stochastic imputation, assuming missing completely at random outcomes, based on the observed mortality of 13.4%

	**Missing outcomes imputed by death** N=896 - deaths=151		**Missing outcomes imputed by alive** N=896 - deaths=120		**Missing outcomes imputed by stochastic imputation** N=896 - deaths=122	
	OR (95% CI)	*p*	OR (95% CI)	*p*	OR (95% CI)	*p*
**Male gender**	1.69 (1.13-2.53)	0.01	1.58 (1.02-2.47)	0.04	1.56 (1.01-2.43)	0.048
**Age (≥60 years)**	1.65 (0.93-2.95)	0.09	1.83 (0.94-3.54)	0.07	1.69 (0.89-3.23)	0.1
**Fatigue**	1.80 (1.16-2.77)	0.008	1.51 (0.93-2.44)	0.09[d]	1.48 (0.92-2.40)	0.1[d]
**Poor performance status (>2)**	2.47 (1.62-3.79)	<0.0001	2.82 (1.79-4.47)	0.00001	2.85 (1.80-4.51)	<0.00001
**Solid malignancy[a]**	2.79 (1.29-6.03)	0.009	2.94 (1.22-7.09)	0.02	3.03 (1.25-7.30)	0.01
**Uncontrolled malignancy**	2.34 (1.49-3.66)	0.0002	2.07 (1.27-3.38)	0.003	2.18 (1.33-3.58)	0.002
**Neurological disorders**	2.08 (1.25-3.49)	0.005	2.17 (1.25-3.78)	0.006	2.29 (1.32-3.97)	0.003
**High Shock-index[b] (≥1)**	1.45 (0.85-2.48)	0.17[c]	1.93 (1.11-3.36)	0.02	1.83 (1.05-3.19)	0.03
**Oxygen therapy**	2.23 (1.45-3.44)	0.0003	2.65 (1.67-4.20)	<0.0001	2.85 (1.80-4.52)	<0.00001

ED emergency departmen*t, OR* odds ratio, *95% CI* 95% confidence interval

[a] Reference: hematological malignancy

[b] Hearth rate/systolic arterial blood pressure

[c] Shock-index was no more associated with the outcome

[d] Fatigue was no more associated with the outcome
